# Using the absolute advantage coefficient (AAC) to measure the strength of damage hit by COVID-19 in India on a growth-share matrix

**DOI:** 10.1186/s40001-021-00528-4

**Published:** 2021-06-24

**Authors:** Daw-Hsin Yang, Tsair-Wei Chien, Yu-Tsen Yeh, Ting-Ya Yang, Willy Chou, Ju-Kuo Lin

**Affiliations:** 1 Department of Gastrointestinal Hepatobiliary, Chiali Chi-Mei Hospital, Tainan, Taiwan; 2 Department of Medical Research, Chi-Mei Medical Center, 901 Chung Hwa Road, Yung Kung Dist, Tainan, 710 Taiwan; 3 Medical School, St. George’s University of London, London, UK; 4 Medical Education Center, Chi-Mei Medical Center, Tainan, Taiwan; 5 School of Medicine, College of Medicine, China Medical University, Taichung, Taiwan; 6 Department of Physical Medicine and Rehabilitation, Chi Mei medical center, Tainan, Taiwan; 7 Department of Ophthalmology, Chi-Mei Medical Center, 700 Tainan, Taiwan

**Keywords:** Four-quadrant diagram, COVID-19, Multiply infection rate, Dashboard, Google maps

## Abstract

**Background:**

The COVID-19 pandemic occurred and rapidly spread around the world. Some online dashboards have included essential features on a world map. However, only transforming data into visualizations for countries/regions is insufficient for the public need. This study aims to (1) develop an algorithm for classifying countries/regions into four quadrants inn GSM and (2) design an app for a better understanding of the COVID-19 situation.

**Methods:**

We downloaded COVID-19 outbreak numbers daily from the Github website, including 189 countries/regions. A four-quadrant diagram was applied to present the classification of each country/region using Google Maps run on dashboards. A novel presentation scheme was used to identify the most struck entities by observing (1) the multiply infection rate (MIR) and (2) the growth trend in the recent 7 days. Four clusters of the COVID-19 outbreak were dynamically classified. An app based on a dashboard aimed at public understanding of the outbreak types and visualizing of the COVID-19 pandemic with Google Maps run on dashboards. The absolute advantage coefficient (AAC) was used to measure the damage hit by COVID-19 referred to the next two countries severely hit by COVID-19.

**Results:**

We found that the two hypotheses were supported: India (i) is in the increasing status as of April 28, 2021; (ii) has a substantially higher ACC(= 0.81 > 0.70), and (iii) has a substantially higher ACC(= 0.66 < 0.70) as of May 17, 2021.

**Conclusion:**

Four clusters of the COVID-19 outbreak were dynamically classified online on an app making the public understand the outbreak types of COVID-19 pandemic shown on dashboards. The app with GSM and AAC is recommended for researchers in other disease outbreaks, not just limited to COVID-19.

**Supplementary Information:**

The online version contains supplementary material available at 10.1186/s40001-021-00528-4.

## Background

Since the outbreak of 2019, novel coronavirus infection (COVID-19) in Wuhan city, China, on 30 January 2020 [1–3], a total of 0.15 billion infections and 3.18 million deaths have been reported as of 28 April 2021. The total number of deaths caused by COVID-19 (2.7 million) has substantially surpassed that of severe acute respiratory syndrome (SARS) (774 deaths in 2003) and the Middle East respiratory syndrome (MERS) (858 deaths in 2012) [4].

As of 28 April 2021, more than 1,28,740 COVID-19-related articles have been released on PubMed Central (PMC) [5]. The Johns Hopkins Center for Systems Science and Engineering (JHC) and other websites have built interesting online dashboards for regularly updated data for tracking the worldwide spread of the COVID-19 outbreak [6–13]. However, those dashboards merely displayed basic information on the numbers of death and confirmed cases of COVID-19 on a world map [14]. None of those dashboards were equipped with more sophisticated knowledge to fulfill the public’s interest and need of the COVID-19 situation, such as using (i) the growth/share matrix (GSM) coined by the Boston Consulting Group (BCG) in 1970 to classify countries/regions in feature (i.e., growth on the Y-axis and share on the X-axis) [15–17], and (ii) the absolute advantage coefficient (AAC) [18–22] to report the strength of damage hit by COIVD-19 when compared to the next two countries/regions (e.g., what are the situations in India using the GSM and the AAC when the deadly second wave of COVID-19 spreads from cities to small towns [23].

### Literature review

#### The GSM coined by the Boston Consulting Group (BCG)

The GSM is the most famous and simple portfolio planning matrix suggested to organizations to achieve a balance between the four categories of products a company produces [24, 25], and was established in 1970 by Bruce Doolin Henderson (1915–1992) for the BCG in Boston, Massachusetts, the USA. Henderson was the President and Chief Executive Officer (CEO) until 1980. He was also Chairman until 1985. The matrix helps the business corporations for the improvement of the skills to run their business efficiently and profitably [26].

To help businesses further analyze their assets, the GSM divides the business products into four categories [17]:

- (i) “Question marks” indicates the products in high growth markets and with low market share (shown in quadrant II),
- (ii) “Stars” shows that both the growth markets and market share are in the highest position (shown in quadrant I),
- (iii) “Cash cows” predicts that the products are in low growth markets, and market share is high (shown in quadrant IV),
- (iv) “Dogs” displays that both growth and market share are in a low position (shown in quadrant III).

We image that all countries/regions hit by COVID-19 are similar to products in GSM. The multiple infection rates (MIR, which is similar to the growth rate of the gross domestic product (GDP) or the multiple interest rate in banking that we are familiar with to denote the growth trend of daily confirmed cases of COVID-19 in the recent one week [27]. The market share in GSM can be represented by the mean MIR of a country/region in COVID-19. As such, we are motivated to apply the GSM to understand the COVID-19 situations for each country/region in the recent seven days. The first hypothesis is whether India is in an increasing quadrant of GSM as of 28 April 2020 when the deadly second wave of COVID-19 spreads from cities to small towns [23].

#### The ACC applied to measure the strength of damage hit by COVID-19

The ACC [18–22] is defined by Eqs. (1) and (2):

1 $${\text{Ratio = }}\frac{{\frac{{\gamma _{{\text{2}}} }}{{\gamma _{{\text{2}}} }}}}{{\frac{{\gamma _{{\text{2}}} }}{{\gamma _{{\text{3}}} }}}},$$

2 $${\text{AAC}} = \frac{{{\text{Ratio}}}}{{{\text{(1 + Ratio}}}},$$

where the ratio is determined by the three consecutive numbers of daily confirmed cases (e.g., total cases in the recent seven days) of countries/regions (i.e., the top three have the most number of confirmed cases) (denoted by *γ*_1_, *γ*_2_,and *γ*_3_ in Eq. (1)). The ACC ranged from 0 to 1.0 stands for the strength of total confirmed cases when compared to the next two countries/regions.

Through the computation of AAC, the strength of damage hit by COVID-19 in India can be measured when compared to the next two following countries/regions.

### The need to display the GSM on a dashboard

Websites targeted toward the public were found to have a various extent of poor to mediocre quality on educational material [28]. As mobile technology continues to expand, assessing health information is common and worthy of continuously improved applications and development for use in epidemic [14, 29–32]. Although numerous COVID-19-related websites [6–13] were globally developed to report the public health risks of the COVID-19, its application and use in displaying the two features of GSM and AAC are lacking. We were motivated to design an app that can provide unique information about COVID-19 situations to the public, particularly using the GSM and AAC to verify whether India has a large effect size of AAC due to the deadly second wave of COVID-19 spreads from cities to small towns [23] as of 28 April 2021.

### Main goals

Based on the two proposed schemes of GSM and AAC, we made two hypotheses that India (i) is in an increasing quadrant of GSM (i.e., continuously increasing), and (ii) has a large effect size of AAC (i.e., greater than 0.70 [20, 33, 34]).

The aims of the current study are to (1) develop an algorithm for classifying countries/regions into four quadrants inn GSM and (2) design an app for a better understanding of the COVID-19 situation.

## Methods

### Data source

We downloaded COVID-19 outbreak numbers for countries/regions on 28 April 2021 from the Github websites [8], which contains confirmed cases in all 189 infected countries/regions, see Additional files 1 and 2. All downloaded data were made available to the public on the websites [8]. Ethical approval is not necessary for this study because all the data were obtained from the websites.

### An algorithm for displaying the growth trend and the mean MIR

#### The MIR definition in growth trend on axil Y

The MIR is based on the geometric mean **(= **${(\prod _{\boldsymbol{i}=1}^{\boldsymbol{n}-1}\frac{{\boldsymbol{X}}_{\boldsymbol{i}+1}}{{\boldsymbol{X}}_{\boldsymbol{i}}})}^{1/(\boldsymbol{n}-1)}-1={\left(\frac{{\boldsymbol{x}}_{\boldsymbol{n}}}{{\boldsymbol{x}}_{1}}\right)}^{\frac{1}{\boldsymbol{n}-1}}-1,$ where x denotes the cumulative count at the last (Xn) and the first (X1) time point, and n stands for the sequentially observed days). In the current study, we calculated the MIR for each country/region on a weekly (= 7 days) basis. For instance, the data string {1,1,1,1,2,4,4,4} of daily confirmed cases for a country yields a MIR of 0.26(= (4/1)^(1/6)−1, where 6 = 7−1).

The growth trend in the spread of COVID-19 is defined as the angle between the two numbers of daily confirmed cases in the period of observed days (i.e., in the recent 7 days). The angle was then computed by Eq. (3):

3 $${\text{Angle index }} = {\mathbf{\theta }} = {\mathbf{Degrees}}\left( {{\mathbf{Atan}}\left( {\frac{{\Delta {\mathbf{CNCC}}_{{\mathbf{k}}} }}{{\Delta {\mathbf{DAY}}_{{\mathbf{k}}} }}} \right)} \right),$$

where Degrees() and Atan() are derived from the functions in Microsoft Excel, and CNCC is the cumulative number of confirmed cases for a county k. For instance, ∆IP = 7 days, ∆CNCC = 1000–600 = 400, ratio = 400/(7 − 1) = 66.6, θ = DEGREES(ATAN(66.6)) = 89.14. The angle index ranges from 0 to 90, wherein a higher θ value means a greater increase of damage hit by COVID-19 in a given country or region.

**Fig. 1:**
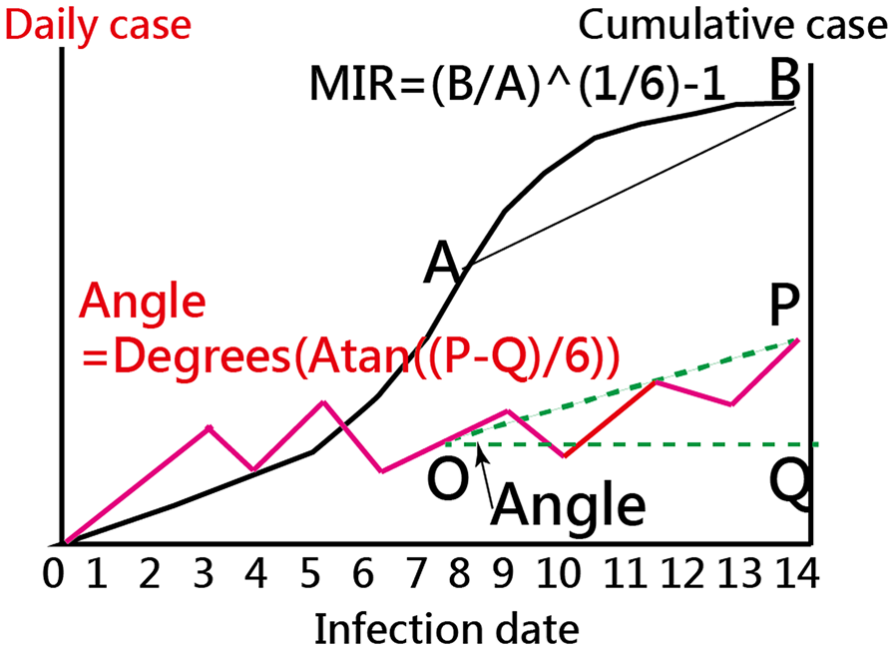
How to compute the MIR and trend (denoted by angle) with visualizations

The explanations of MIR (interpreted by the geometric mean) and trend (denoted by angle) are shown in Fig. 1. We can see that the MIR equals zero when the stationarity is present owing to the two points (e.g., A and B in Fig. 1) being identical (e.g., 210 and 210 in CNCC). The trend is denoted by the angle using the formula of Degrees (Atan((P-Q)/6) in MS Excel, ranging between − 90 to 90.

#### The mean infection rate (MIR) as the share on axil X

The X-axis (trend magnitude) is based on the geometric mean MIR in the recent seven days. The Y-axis (trend growth) is derived from the angle from − 90 to 90 mentioned above. As such, the four-quadrant diagram in GSM was applied to classify countries/regions into four clusters (i.e., ready to increase, increasing, slowing down, and ready to decrease) [17].

**Fig. 2:**
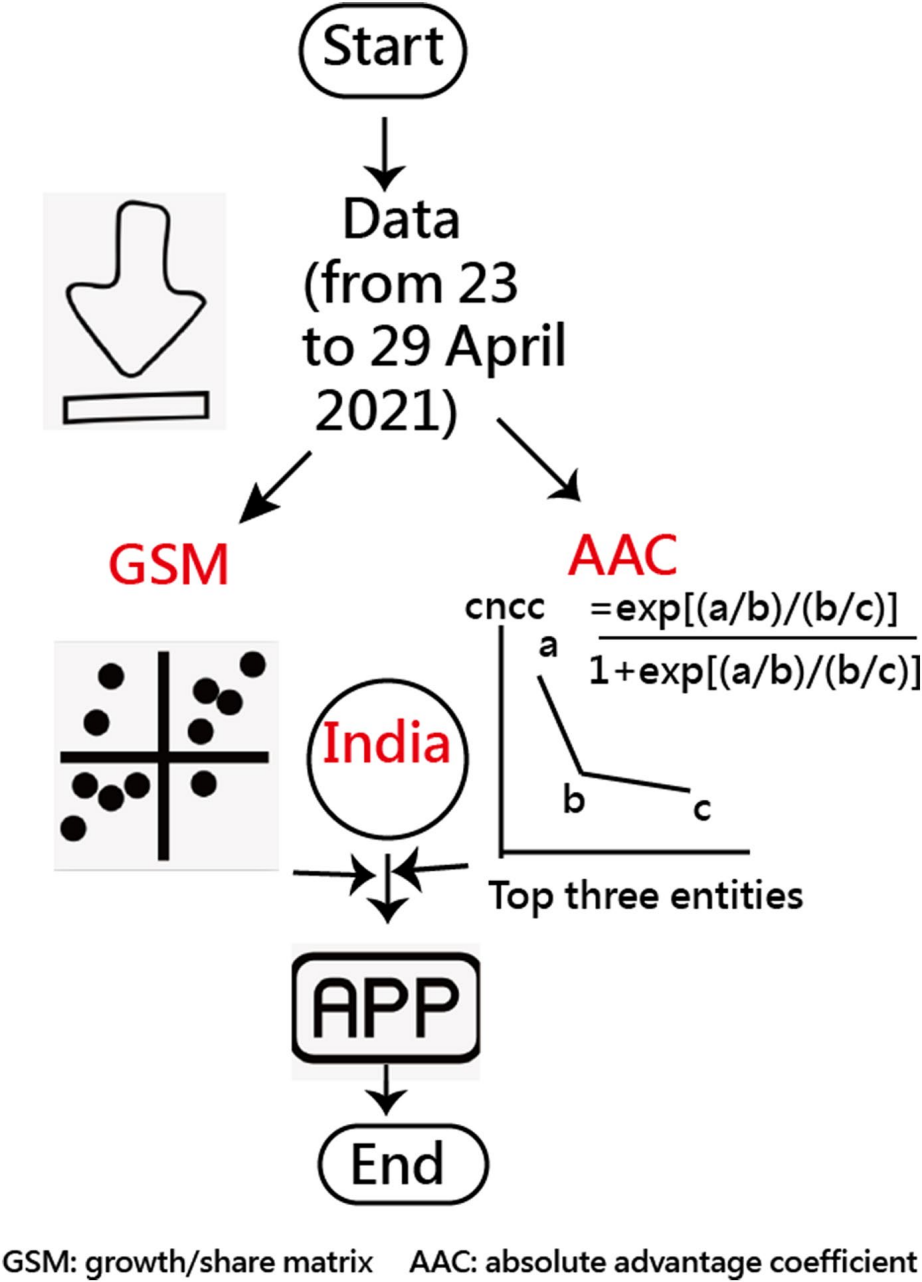
The study design is composed of two scenarios and two axes to classify entities into four clusters

The absolute cutting points on Y-axis are determined at 0 degrees, and the relative cutting point on X-axis is determined at the median of all Mean MIRs computed from the 189 study countries/regions. The first hypothesis that India is in Quadrant I of GSM would be verified. The flowchart and the abstract video of this study are provided in Fig. 2 and Additional file 3.

#### AAC to measure the strength of damage hit by COVID-19 related to the next two countries

The AAC is defined in Eq. (2). India was demonstrated to compute the AAC when compared to the following two countries severely hit by COVID-19 based on the CNCC than India as of 28 April. 2021. The second hypothesis that India has a large effect size of AAC(> 0.70) would be verified in this study. Notably, If all those total daily cases are equal in the recent 7 days, the AAC is 0.5(= [(1/1)/(1/1)]/(1 + [(1/1)/(1/1)]) = 1/2 = 0.5.

### App classifying growth trends and MIRs for countries/regions

Based on the daily reports from the Github website [8], we built an updated online dashboard for tracking the worldwide spread of the COVID-19 outbreak with data collected as of 28 April 2021.

### Creating dashboards on google maps

The classifications for each country/region were shown by author-made online modules. We created HTML pages for Google Maps. All the relevant COVID-19 information on the countries/regions can be linked to dashboards on Google Maps. Bubbles were sized and colored by (1) the cumulative daily number of confirmed cases in the recent 7 days and (2) the cluster feature in GSM, respectively. When a specific bubble is clicked, further information and hyperlinks to the trend chart appear on the dashboard.

## Results

### An overall view on a choropleth map

**Fig. 3:**
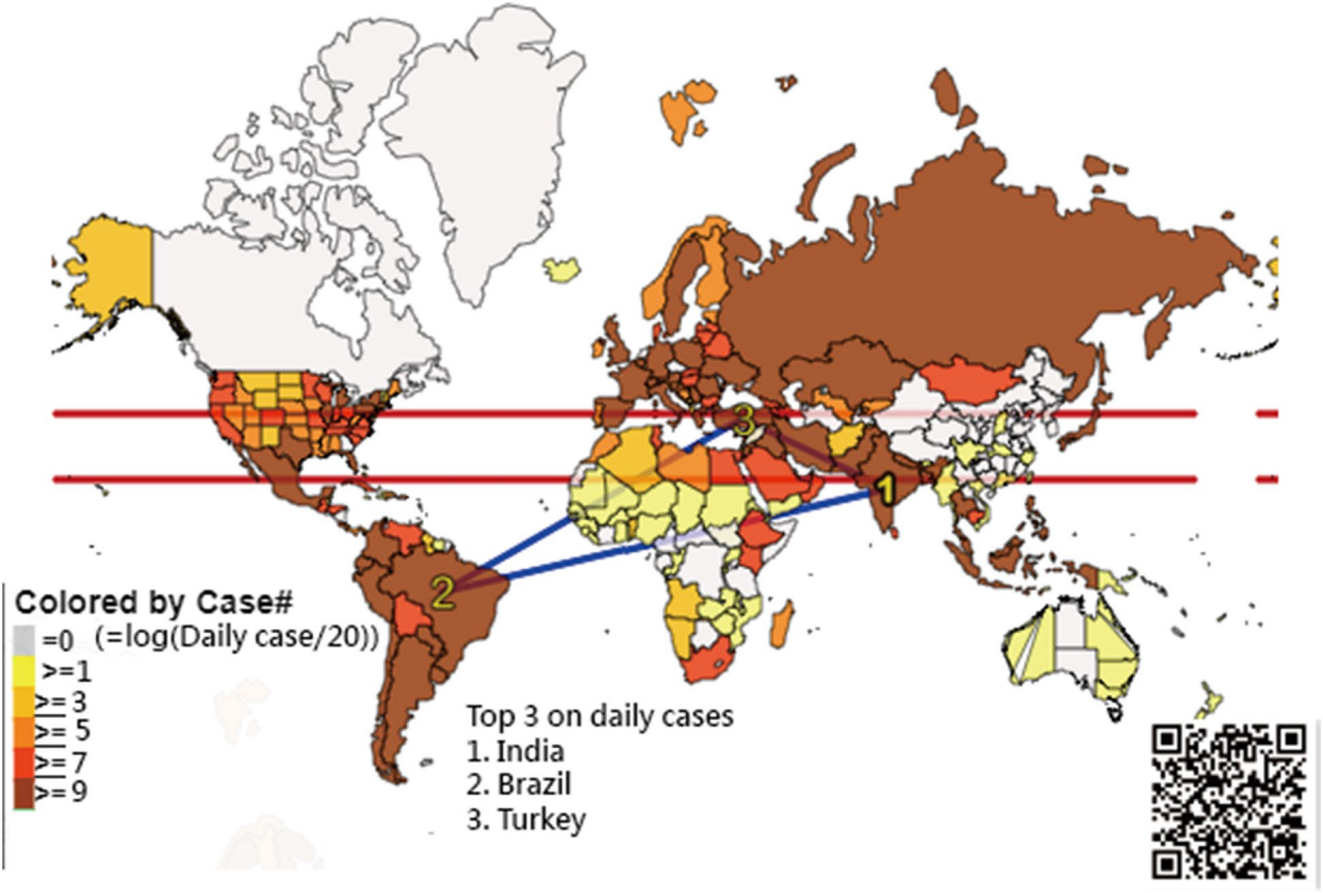
The recent daily confirmed cases shown on the choropleth map as of 28 April 2021

Comparisons in daily confirmed cases of COVID-19 were made using the choropleth map in Fig. 3. The top three countries are India (= 3,79,308 per day), followed by Brazil (= 79,726) and Turkey (= 40,444). The AAC based on the daily confirmed cased on 28 April 2021 is 0.71(= (379308/79726)/(79726/40444)/[1 + (379308/79726)/(79726/40444)].

Readers are invited to practice the visual representation on Google Map via the link [35] or the QR-code in Fig. 3.

**Fig. 4:**
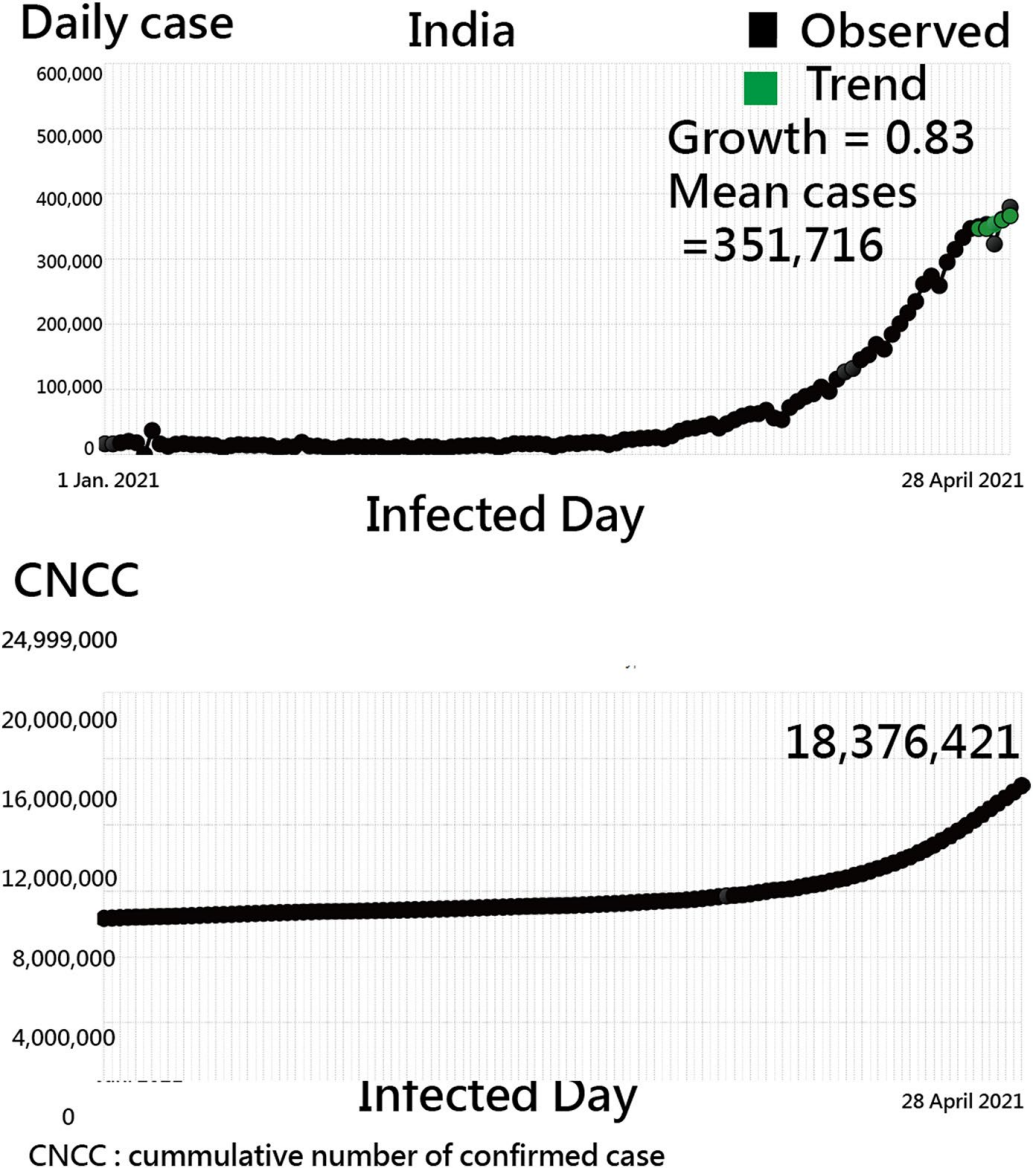
Line plots for India as of 28 April 2021 to present the trend when observing daily and CNCC cases since 1 January 2021(Note. the trend is determined by the green dots connected by the first and the end dots in the recent 7 days)

If the colorful region is clicked, two-line plots immediately appear on a dashboard. The example of India is demonstrated as of 28 April 2021 in Fig. 4. The trend was observed via the daily and the CNCC cases since 1 January 2021.

### The GSM is shown on a dashboard

The COVID-19 GSM is shown on a dashboard. Bubbles are sized by the mean daily cases in the recent seven days and colored by the features of growth and share in GSM. That is, yellow bubbles in Quadrant II represent the pandemic situation ready to increase (e.g., Israel), green bubbles in Quadrant I stand for the situation increasing (e.g., India), red bubbles in Quadrant III mean the situation slowing down (e.g., New Jersey in US), and purple bubbles are ready to decrease in Quadrant IV (e.g., Turkey and Thailand). It is worth noting that the growth and share are denoted on axil Y and axil X, respectively.

**Fig. 5:**
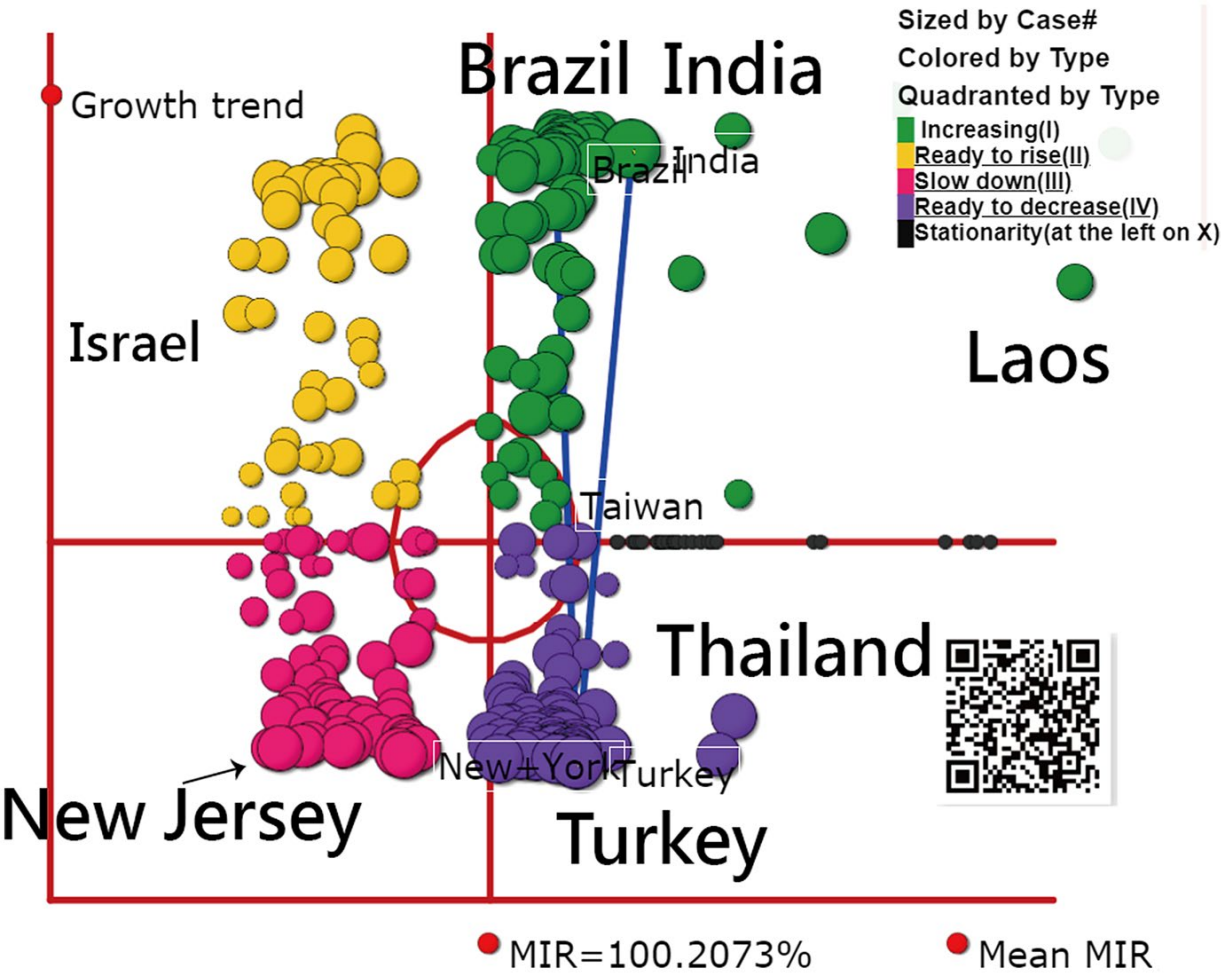
The GSM of COVID-19 based on bubbles sized by the mean daily cases and colored by the features of growth and share shown on a dashboard

As expected, top three countries of India, Brazil, and Turkey are highlighted by three blue lines linked together in Fig. 5. The first hypothesis that India is in the increasing quadrant of GSM is supported in Fig. 5.

**Fig. 6:**
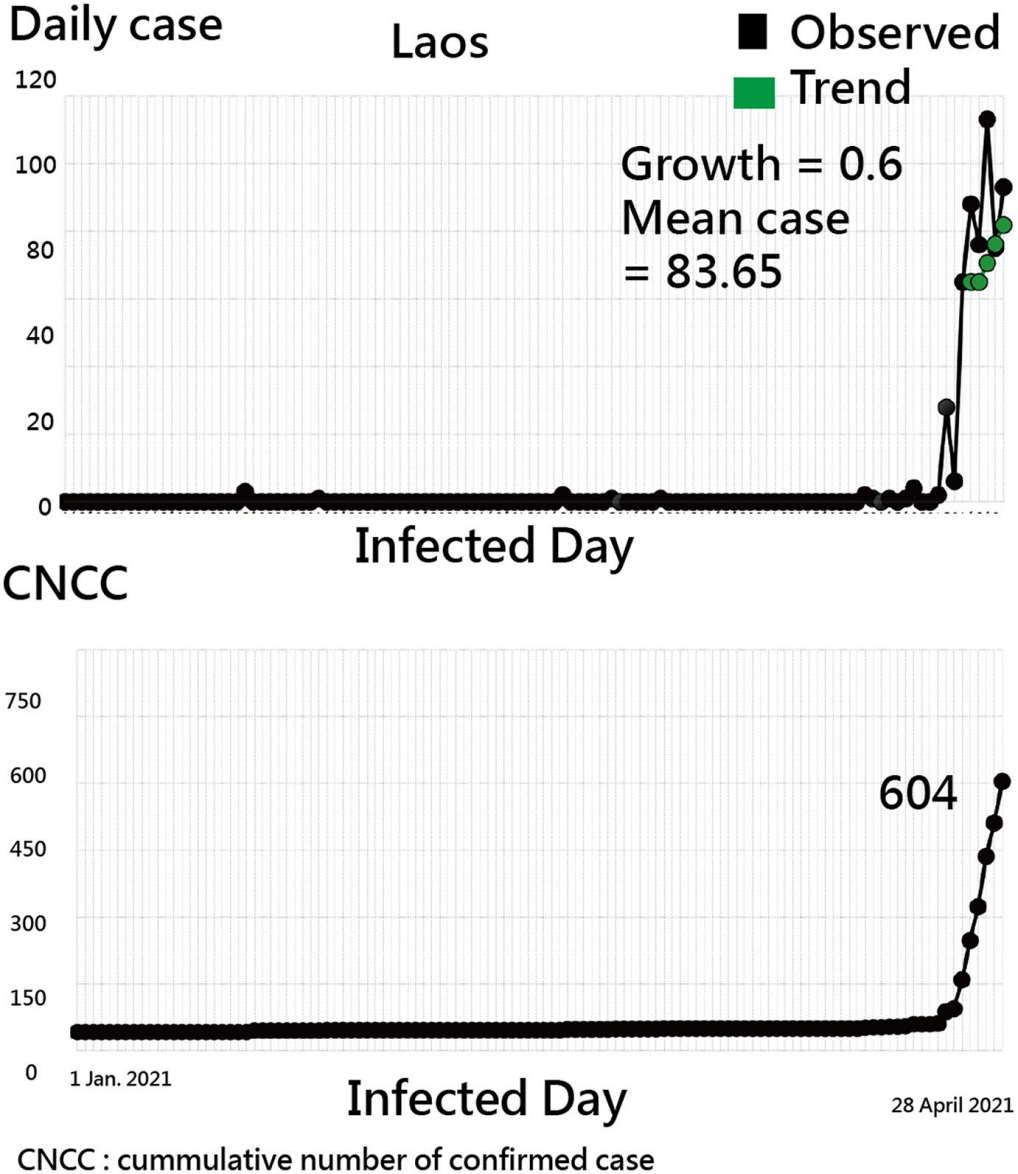
Line plots for Laos as of 28 April 2021 to present the trend when observing daily and CNCC cases since 1 January 2021. (Note the trend is determined by the green dots connected by the first and the end dots in the recent seven days)

Surprisingly, Laos was neglected by the public when observing the GSM in Fig. 5. If the line plots are drawn as shown in Fig. 6, the higher growth and share (denoted by MIR) in Laos can be easily seen. Accordingly, the outliners are easily examined through the GSM, as we demonstrated in Fig. 5.

Readers are invited to practice the visual representation on Google Map via the link [36] or the QR-code in Fig. 5.

### The AAC of India

The AAC of India is 0.81(= (2112726/353316)/(353,316/249644)/(1 + 2,112,726/353316)/ (353316/249644)) because the total daily cases in the recent 7 days for the top three countries of India, Brazil, and Turkey are 2,112,726, 353,316, and 2,49,644, respectively. The second hypothesis that India has a large effect size of AAC (i.e., greater than 0.7 [20, 33, 34]) was supported (i.e., 0.81 > 0.7). If the day is extended to 1 May 2021, the AAC of India is 0.79.

If AAC(= 0.54) for Laos followed by Guangxi (China) and Shanxi (China) is computed, the formula can be composed of (2374/1890)/(1890/1744)/[1 + (2374/1890)/(1890/1744)].

### Online dashboards shown on google maps

**Fig. 7:**
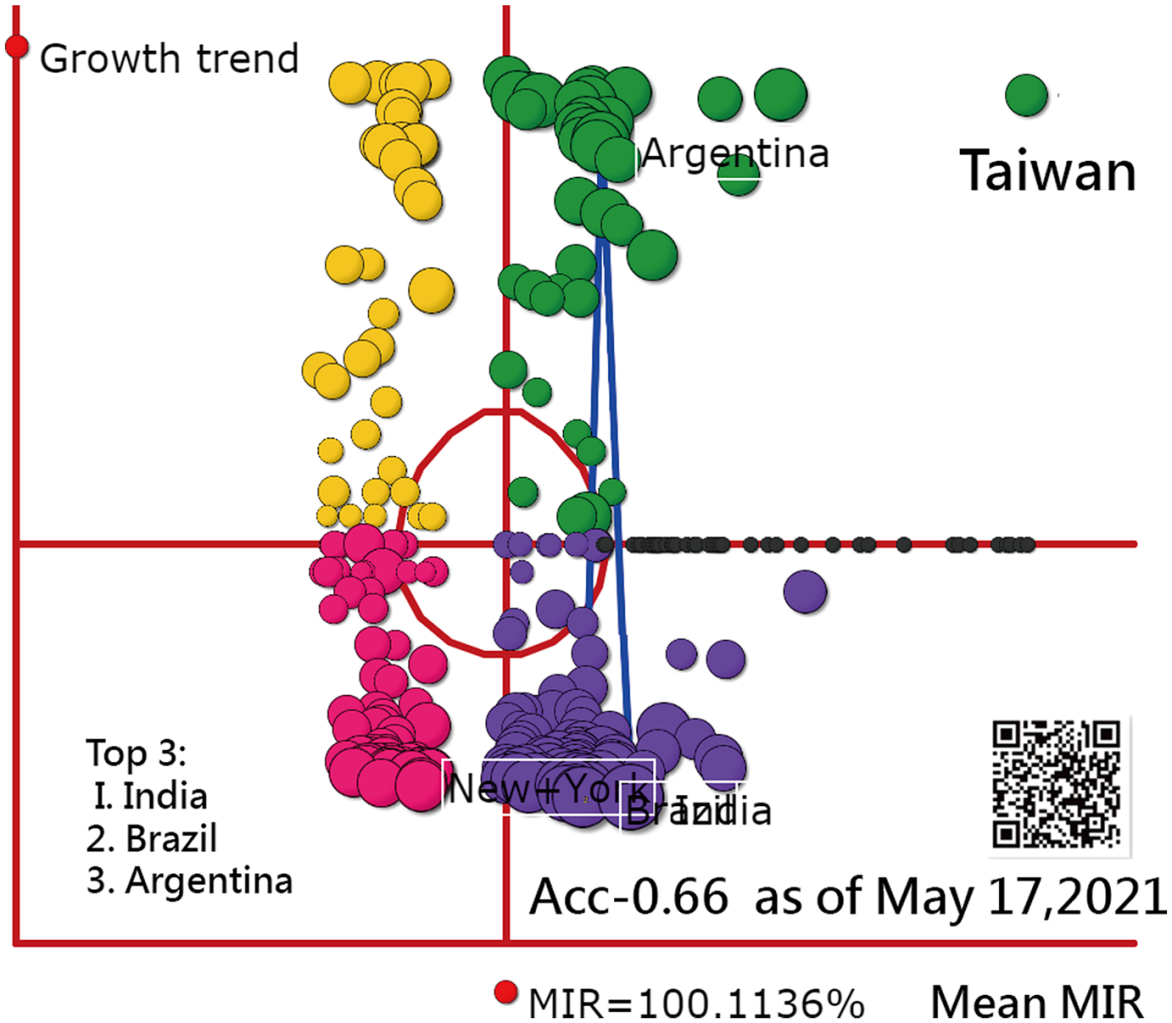
The 4-Q diagram as of May 17, 2021 from 0.81 on April 28, 2021

All of those QR codes in the figures are linked to the dashboards [35, 36]. Readers are recommended to examine the displayed dashboards on Google Maps. The 4-Q diagram as of May 17, 2021, is present in Fig. 7. We can see that Taiwan was severely hit by COIVD-19. The top 3 countries with most number of confirmed cases in recent seven days were India, Brazil, and Argentina. The ACC has down to 0.66.

## Discussion

### Principal findings

**Fig. 8:**
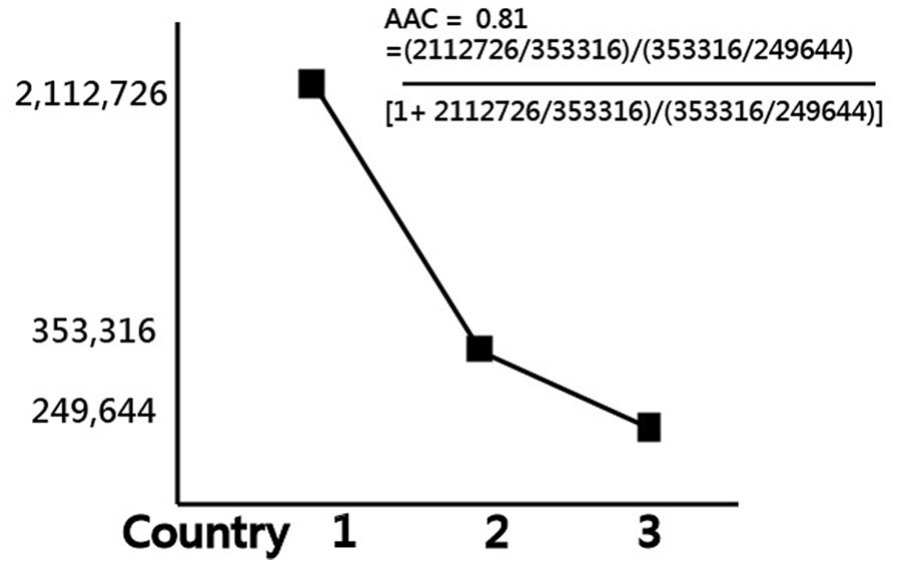
The calculation of AAC for India as of April 28, 2021

Based on the data of COVID-19 as of 28 April 2021, the two hypotheses were supported: (i) India is in the increasing quadrant of GSM in Fig. 5, and (ii) India has a substantially larger effect size of AAC(= 0.81 > 0.7) shown in Fig. 8.

The two goals were also achieved by (1) developing an algorithm for classifying countries/regions into four quadrants in GSM and (2) designing an app for a better understanding of the outbreak situation of COVID-19.

### Contributions of the study

#### The GSM

This study extends the Boston Consulting Group four-cell growth/share product portfolio matrix [24, 25]. The BCG matrix was originally applied to products; however, in this case, the BCG concept is applied to countries/regions hit by COVDI-19, which were classified into four quadrants based on the growth (denoted by the angle of CNCC in the recent seven days) on the Y-axis and the share (denoted by the mean MIR) on the X-axis. In this study, we verified that the COVID-19 situation in India is increasing and has a substantially higher AAC(= 0.81) when compared to the two next following countries with the most number of CNCC, indicating India is most severely hit by COVID-19.

The GSM is based on the CNCC growth and share represented by the angle in Eq. (3) and the magnitude of mean MIR. If a country/region has a steeper trend and a bigger magnitude in MIR, there will be more infections from the disease outbreak than other countries/regions. The BCG matrix was thus applied to measure the ongoing outbreaks in this study, the type of hospitals in therapeutic duplication for patients with high blood lipids [17], the relative importance of each customer to the company's total profit by segmenting customers into portfolios [37], the smart farms classified in the Supply Chain to supplement the revenues of their underlying product sales [38], and the export competitiveness of Malaysia processed food in the middle east market [39].

The classification has been used by humans for thousands of years. It is also important to our everyday life and applies to almost everything we do, allowing us to find and recognize things more easily [40, 41]. Imagine if we went to a library without classification—where would we start looking for a particular book? Similarly, if we encountered the COIVD-19 spreads around the world without classification—where would we start looking for mitigating the impact of the COVID-19 pandemic on progress towards ending the COIVD-19 spreads.

#### The AAC

Except for the GSM, AAC was applied to measure the strength of damage in a country/region hit by COVID-19 when compared to the next following two countries. Under the influence of a pandemic like COVID-19, we hoped to develop an index that can determine the dominant roles in countries/regions [19], which is similar to that used in determining the strength of the leading company in an industry using the separation index [42] and the Herfindahl index (HI) proposed by economists Orris C. Herfindahl and Albert O. Hirschman [43] to investigate the competition in the industry [44].

The same applied to what we investigated one questionnaire (or test) as a unidimensional construct is using Eigenvalues [20] to determine the dimension coefficient with the cutting point at 0.70 as we did in Eq. (2). Furthermore, the most cited article (PMID = 23563266 with 2604 citations in PMC) [45] with a higher AAC(= 0.77) in article citations in the fields of dengue fever [46].

#### The mobile APP

Additionally, numerous websites have built online dashboards for regularly updated data for tracking the worldwide spread of the COVID-19 outbreak [6–13]. Almost all of them merely provided common COVID-19-related information to the public [14]; see the snapshot from JHC in Additional files 2 and 3. A dashboard-type interface with useful information is required to provide the public with an interpretation of COVID-19 (or other pandemic diseases) with the four classifications in color, particularly for mobile apps in our modern technological age, as we did in this study.

### Strengths and implications in this study

The first feature is to demonstrate the four-quadrant diagram of GSM and its relevant line charts with MP4 video in Additional files 2 and 3. The dashboard-type GSM can be further applied to other disease outbreaks in the future, not limited to COVID-19.

The two topics of (i) GSM and (ii) AAC for India were demonstrated in this study. We can see that the outliers in GSM are easily examined (e.g., Laos found in Figs. 5, 6). The four-quadrant diagram of GSM displayed on the dashboard is innovative. We have not seen a similar dashboard combined with the growth and the share (denoted by the mean MIR) to show the COVID-19 situation in the literature.

As with all forms of Web-based technology, advances in health communication technology are occurring every moment [47]. The real-time mobile online dashboard for COVID-19 provided to the public is the fourth feature. Dashboards with four quadrants are practical and worth replicating for other disease outbreaks. The mobile online dashboard is informative and has value in keeping the public up-to-date, especially during a disease outbreak, much such as the one we are facing now. Readers are recommended to scan the QR codes on the figures and see the details about the outbreak trends using the line charts [35, 36].

### Limitations and suggestions

Our study has some limitations. First, although the data were downloaded from Google Sheets daily, the online near real-time dashboard could be improved and polished by connecting to Google Sheets instantly. Other dashboard features, such as the color selections, the app layout design, and employed predictive analytics [48–52], should be improved in the future.

Second, although the novel visual representations (Figs. 4, 5) were proposed in this study, the bubble of interest can be linked to more information about the details of the countries/regions with the improved app on performance and features in the future.

Third, many innovations have been introduced with advances in science and technology, such as the visual dashboard on Google Maps using the coordinates to display and line plots on cloud computation, as shown in Figs. 3, 4, 5. However, these achievements are not free of charge. For example, the Google Maps API requires a paid project key for use on the cloud platform, and the line plot also requires payment (to JPowered) for the template used on the website. Thus, the second limitation of the module is that it is not publicly accessible and is difficult to mimic by other authors or programmers for use in a short period of time.

Fourth, the mascots were applied to the traditional GSM in BOG24,25], such as stars, problem children, cash cows, and dogs, that might be inappropriate in health care settings (e.g., the entries are now countries hit by COVID-19 rather than products in the original GSM [24, 25]). Appropriate mascots, such as platinum, gold, silver, bronze, and iron denoted by colors of black, green, yellow, purple, and red in GSM, respectively, might be applied to the feature of quadrants in GSM with regard to COVID-19.

Finally, the GSM is constructed by growth and share. The latter is formed by the mean MIR (i.e., the infection rate in the recent 7 days) instead of the CNCC traditionally used in the epidemic. Although the CNCC is sized by the bubble in GSM, the CNCC on the X-axis could be attempted in the future. If so, India would be on the far right side and Laos on the left side. The infection rate would not be highlighted in GSM.

#### Conclusion

We included subtle algorithms in an app that makes the COVID-19 information more useful and meaningful to the general public. Readers who are interested in the four-quadrant diagram of GSM are invited to link the website with the QR codes in figures. More useful messages provided to the public are required to develop particular algorithms on an app in the future. The app with GSM and AAC is recommended for researchers in use in other disease outbreaks, not just limited to COVID-19.

## Supplementary Information

**Additional file 1**. Data and images at https://osf.io/2zhgv/?view_only=a8523ab3c23944b19096e8272d1846a0(accessed on 17 May2021).

**Additional file 2**. The process of downloading data from Github at https://youtu.be/nO4VxjyxFqM (accessed on 17 May 2021).

**Additional file 3**. MP4 video of this study at https://youtu.be/7J61VMVfZ60 (accessed on 17 May 2021).

## Data Availability

All data used in this study are available in Supplemental Digital Contents. Additional file 1, 2 ,3.

## Abbreviations

BCG: Boston consulting group
GDP: Gross domestic product
MIR: Multiple infection rate
PMC: Pubmed Central
JHC: The Johns Hopkins
